# Cinacalcet lowering of serum fibroblast growth factor-23 concentration may be independent from serum Ca, P, PTH and dose of active vitamin D in peritoneal dialysis patients: a randomized controlled study

**DOI:** 10.1186/1471-2369-14-112

**Published:** 2013-05-25

**Authors:** Hyo Jin Kim, Hyunsuk Kim, Nara Shin, Ki Young Na, Yong Lim Kim, Daejung Kim, Jae Hyun Chang, Young Rim Song, Young-Hwan Hwang, Yon Su Kim, Curie Ahn, Joongyub Lee, Kook-Hwan Oh

**Affiliations:** 1 Department of Internal Medicine, Seoul National University, Seoul, Korea; 2 Department of Internal Medicine, Seoul National University Bundang Hospital, Seongnam-si, Gyeonggi-do, Korea; 3 Department of Internal Medicine, Kyungpook National University, Daegu, Korea; 4 Department of Internal Medicine, Sungkyunkwan University, Seoul, Korea; 5 Department of Internal Medicine, Gachon University, Incheon, Korea; 6 Department of Internal Medicine, Hallym University, Seoul, Korea; 7 Department of Internal Medicine, Eulji University, Seoul, Korea; 8 Medical Research Collaborating Center, Seoul National University Hospital, Seoul, Korea

**Keywords:** Cinacalcet, Fibroblast growth factor 23, Peritoneal dialysis

## Abstract

**Background:**

Elevated serum level of fibroblast growth factor-23 (FGF23) is associated with adverse outcomes in dialyzed patients.

**Objectives:**

The CUPID study compared the efficacy of a cinacalcet-based regimen with conventional care (vitamin D and P binders) for achieving the stringent NKF-K/DOQI targets for peritoneal dialysis (PD) patients. Additionally, we analyzed change in FGF23 levels between two treatments to explore the cinacalcet effect in lowering FGF23.

**Design:**

Multicenter, open-labeled, randomized controlled study.

**Setting:**

Seven university-affiliated hospitals in Korea.

**Participants:**

Overall, 66 peritoneal dialysis patients were enrolled.

**Intervention:**

Sixty six patients were randomly assigned to treatment with either cinacalcet + oral vitamin D (cinacalcet group, n = 33) or oral vitamin D alone (control group, n = 33) to achieve K/DOQI targets. CUPID included a 4-week screening for vitamin D washout, a 12-week dose-titration, and a 4-week assessment phases. We calculated mean values of iPTH, Ca, P, Ca x P, during assessment phase and final FGF23 to assess the outcome.

**Main outcome measures:**

Achievement of >30% reduction of iPTH from baseline (primary) and FGF23 reduction (secondary).

**Results:**

72.7% (n = 24) of the cinacalcet group and 93.9% (n = 31) of the control group completed the study. Cinacalcet group received 30.2 ± 18.0 mg/day of cinacalcet and 0.13 ± 0.32 μg/d oral vitamin D (*P* < 0.001 *vs.* control with 0.27 ± 0.18 μg/d vitamin D). The proportion of patients who reached the primary endpoint was not statistically different (48.5% *vs.* 51.5%, cinacalcet *vs.* control*, P* = 1.000). After treatment, cinacalcet group experienced a significant reduction in FGF23 levels (median value from 3,960 to 2,325 RU/ml, *P* = 0.002), while an insignificant change was shown for control group (from 2,085 to 2,415 RU/ml). The percent change of FGF23 after treatment was also significantly different between the two groups (− 42.54% *vs.* 15.83%, *P* = 0.008). After adjustment, cinacalcet treatment was independently associated with the serum FGF23 reduction.

**Conclusion:**

Cinacalcet treatment was independently associated with the reduction of FGF23 in our PD patients.

**Trial registration:**

Controlled trials NCT01101113

## Background

Fibroblast growth factor-23 (FGF23) is an endocrine hormone that regulates phosphate and vitamin D homeostasis. FGF23 is synthesized and secreted by bone cells, mainly osteocytes and plays a role as a phosphatonin by binding to klotho/FGF-receptor complex [1,2]. FGF23 inhibits phosphate reabsorption in the renal tubule and promotes phosphaturia by down-regulating sodium-phosphate co-transporters [3]. It decreases renal production of 1,25(OH)_2_D by inhibiting 1α-hydroxylase and up-regulating 24-hydroxylase in the proximal tubule [3,4]. As kidney function decreases in chronic kidney disease (CKD) patients, FGF23 increases progressively in order to regulate phosphate homeostasis [5]. In advanced CKD, FGF23 no longer maintains phosphate homeostasis and the suppression of 1,25(OH)₂D production exerted by FGF23 induces PTH elevation, resulting in secondary hyperparathyroidism (SHPT). Several studies reported that high levels of FGF23 are related to left ventricular hypertrophy [6-9] and progression of chronic kidney disease [10]. Furthermore, FGF23 elevation is associated with increased mortality in CKD and ESRD patients [11]. Therefore, in CKD patients, modulation of FGF23 could possibly be a therapeutic target in preventing morbidity and mortality. Cinacalcet hydrochloride is a novel calcimimetics that acts by allosteric binding to the calcium sensing receptor on the parathyroid cell membrane and thus lowering PTH. To date, several studies reported that cinacalcet reduced FGF23 in predialysis or hemodialysis patients [12,13].

It was shown that FGF23 levels are independently correlated with serum concentrations of P, Ca x P, and serum parathyroid hormones (PTH). Recently, a few clinical trials on the efficacy of cinacalcet on the lowering of PTH exhibited that cinacalcet treatment lowers FGF23 in ESRD patients [12,13]. However, above studies have not elucidated whether FGF23 suppression during cinacalcet treatment is mediated indirectly via the modulation of serum P and PTH or directly by cinacalcet *per se*. The aim of the Cinacalcet study for Peritoneal Dialysis Patients In Double Arm on the Lowing Effect Of iPTH Level (CUPID) Trial (NCT01101113, registered at the http://www.clinicaltrials.gov) is to investigate the effectiveness of a cinacalcet-based regimen versus vitamin D based regimen in treating SHPT, controlling PTH, Ca, and P levels for achieving the K/DOQI targets for peritoneal dialysis (PD) patients. Additionally, we also investigated whether the cinacalcet effect on the FGF23 suppression occurs directly, independent of its modulation of Ca, P, PTH and exposure to vitamin D.

## Methods

### Study population

Seven university-affiliated hospitals in Korea participated in the CUPID trial. Patients were considered for the study if they were ≥18 and ≤70 years of age, had received peritoneal dialysis (PD) for ≥3 months and with pre-screening intact PTH (iPTH) ≥300 pg/ml (≥250 pg/ml if they had been taking vitamin D analogs) and albumin–corrected calcium ≥9.0 mg/dl. After a 4-week washout period during which vitamin D was withheld, iPTH and calcium levels were measured again. Patients with post-washout iPTH ≥300 pg/ml and corrected calcium ≥9.0 mg/dl were finally enrolled in the study.

Patients were excluded if they had acute inflammatory disease within 3 months, chronic inflammatory disease, gastrointestinal bleeding within 3 months, and were pregnant or breast feeding. Patients who had been taking azole antifungal agents, macrolide antibiotics, amiodarone, tricyclic antidepressant, vinca alkaloid, bupropion antipsychotics, calcitonin, biosphosphonate, steroid hormones, or benzodiazepine drugs were excluded. The study adhered to the principles of the Declaration of Helsinki. The independent ethics committee for each participating center (Seoul National University Hospital Institutional Review Board; Seoul National University Bundang Hospital Institutional Review Board; Kyungpook National University Hospital Institutional Review Board; Samsung Medical Center Office of Research Subjects Protection; Gachon University Gil Medical Center Institutional Review Board; Hallym University Sacred Heart Hospital Institutional Review Board/Ethics Committee; Eulji University Hospital Institutional Review Board) approved the study protocol, and all patients provided written informed consent.

### Study design and treatments

**Figure 1:**
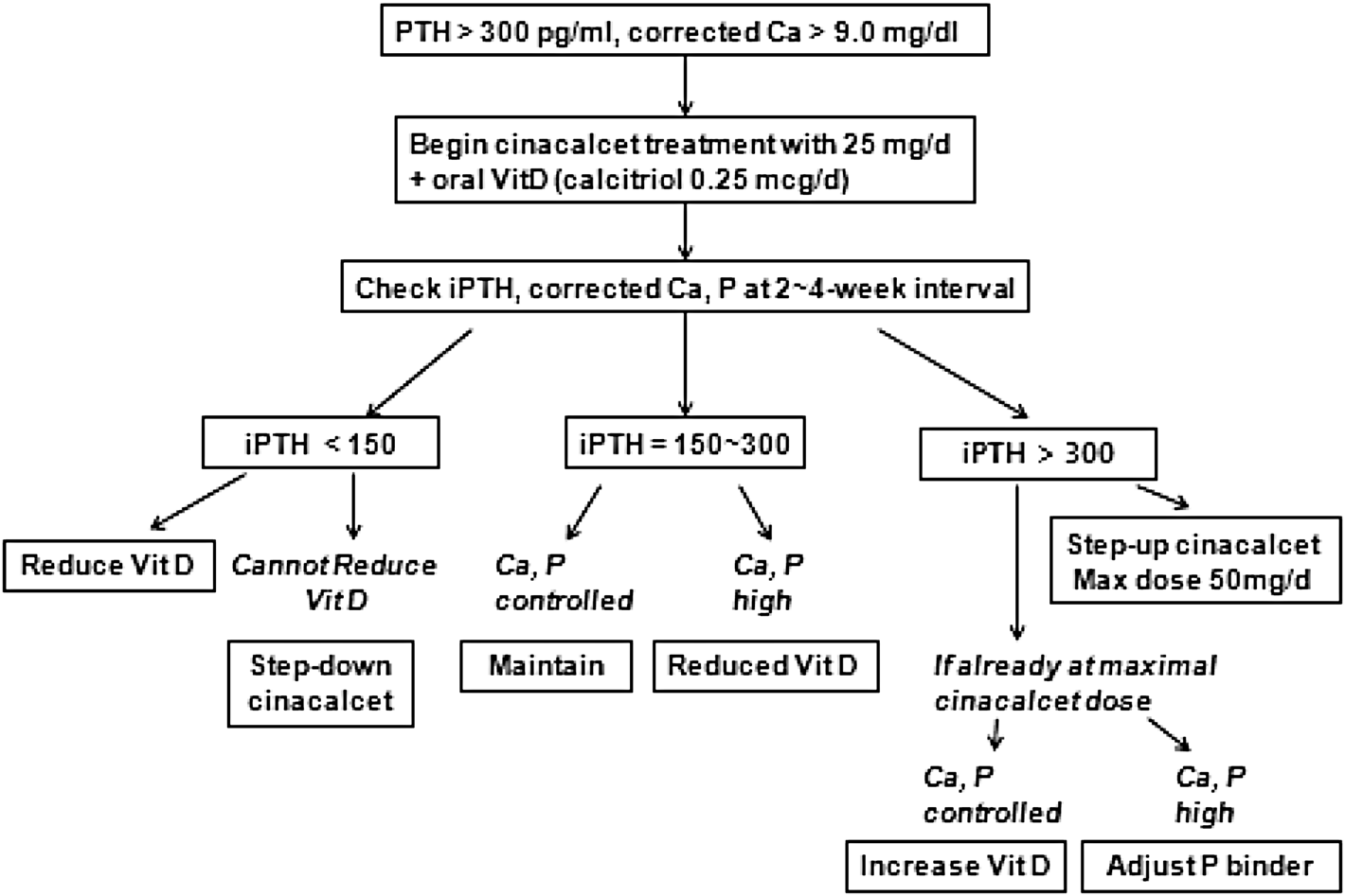
**Dose titration algorithm for cinacalcet and vitamin D in the cinacalcet group during dose-titration period.** Initially, cinacalcet group started with 25 mg/day of cinacalcet p.o. and the cinacalcet dose was, in a stepwise pattern, increased at a 4-week interval to 50 mg/day to achieve an iPTH target between 150 and 300 pg/ml. iPTH, intact parathyroid hormone; Ca, calcium; P, phosphorus; Vit D, vitamin D.

This is a multicenter, open-labeled, and randomized controlled study fulfilled at seven sites. The 20-week study consisted of three phases: a 4-week screening and vitamin D washout phase, a 12-week dose-titration phase, and a 4-week efficacy-assessment phase. At the end of 4-week screening phase, patients were randomly assigned at 1:1 ratio to receive either cinacalcet-based or vitamin D-based therapy. The specific prescription for the cinacalcet group was adjusted according to the treatment algorithm (Figure 1). No subjects had ever taken cinacalcet before screening since cinacalcet was not available in Korea at the time of the study. After screening and vitamin D washout phase, cinacalcet group started with 25 mg/day of cinacalcet (Regpara; Kyowa Hakko Kirin, Co., Ltd, Tokyo, Japan) p.o. and the cinacalcet dose was, in a stepwise pattern, increased at a 4-week interval to 50 mg/day to achieve an iPTH target between 150 and 300 pg/ml. There was no dosage increase within 4 weeks after starting a 25 mg dose, or if the corrected Ca was ≤8.4 mg/dL. The cinacalcet dose was to be reduced if iPTH <150 pg/ml and the dose of concomitant vitamin D could not be further reduced. Cinacalcet were withheld if corrected Ca ≤7.5 mg/dL, or the subjects had symptomatic hypocalcemia, or experienced severe adverse reactions. After starting cinacalcet, the dose of vitamin D prescription was adjusted by treatment algorithm to attain serum iPTH, Ca, and P targets. Vitamin D dosage was reduced if iPTH <150 mg/ml or if iPTH between 150 and 300 with corrected Ca ≥10.2 mg/dL or P ≥5.5 mg/dL.

The control group received flexible dose of oral active vitamin D to achieve K/DOQI targets for PTH, Ca, P, and Ca × P. For active vitamin D, only oral calcitriol, not active vitamin D analogs, was prescribed for both groups. Phosphate binders were allowed to be prescribed at the physician’s discretion throughout the study for both groups. The subjects were not allowed to change dialysate calcium concentration during the entire study period and could not change cinacalcet and vitamin D dosage during the efficacy-assessment phase.

### Biochemical determinations and FGF23

PTH was measured by intact PTH assays at 4-week intervals. All centers were instructed to use the same type of PTH assay throughout the entire study period. Serum Ca, P, and albumin level were measured every 4 weeks but measured twice at 2-week interval if the dose of cinacalcet was changed. ‘Baseline values’ of the various biochemical tests – Ca, P, iPTH, etc. - were defined as those measured after washout period (post-washout).

FGF23 levels were determined at baseline (post-washout) and at the end of assessment phase. Second generation human FGF23 (C-terminal) ELISA kit (Immutopics, San Clemente, California, USA) was employed. The serum was diluted ten-fold with dilution reagent to measure C- terminal FGF23, since the assay accuracy was not validated above this dilution level. The assay range was between 1.5 and 14,900 RU/ml. Over-range of FGF23 which could be not detected after dilution, were excluded in the analysis: 2 patients in the cinacalcet group at baseline; 1 in the control group at baseline; 1 in the control group at efficacy assessment phase.

### Study end points

The mean values of corrected Ca, P, and corrected Ca × P during assessment period were calculated to analyze the results. The primary endpoint was the achievement of >30% reduction of iPTH from baseline. The secondary end points were the absolute or percent change of FGF23 from baseline and the achievements of the K/DOQI targets for the iPTH (150~300 pg/ml), Ca (8.4 ~ 9.5 mg/dL), P (3.5 ~ 5.5 mg/dL), Ca x P (<55 mg^2^/dL^2^), and composite of above targets. We estimated target enrollment to be 85 subjects (based on α= 0.05, β=0.2, and 10% drop-out rate) in order to prove more than 32% difference between the treatment and control arms in the achievement of the primary end point [14].

### Statistical analysis

Baseline demographic and mineral metabolic parameters were analyzed: differences in gender and underlying diseases between treatment groups were evaluated with *χ*^2^ tests; patient’s age, duration of dialysis, and baseline parameters were evaluated with independent samples *t*-tests. Results are presented as mean ± SD for normally distributed variables and median [interquartile range (IQR)] for variables with skewed distributions. A log and square root transformation was used to normalize variability of the iPTH and FGF23 data distribution, respectively; log and square root transformation were implemented before entry into analysis. The demographic variables, percent change in Ca, P, log-transformed iPTH, and the vitamin D dose were used for univariate analysis. Variables showing a significant association (P <0.01) in the univariate analysis or of considerable theoretical relevance were retained as potential predictors in the multivariate model (enter method). Percent change measures were chosen for analysis to alleviate the biasing effect of the patient’s baseline mineral metabolic levels. The percent change was calculated for each mineral metabolic parameter with the following formula: [(week 16 measurement – baseline measurement)/baseline measurement] × 100. Percent changes of the log iPTH and the square root FGF23 were calculated similarly: [(log week 16 measurement – log baseline measurement)/log baseline measurement] × 100; [(square root week 16 measurement – square root baseline measurement)/square root baseline measurement] × 100. Whether or not the pretreatment (post-washout) FGF23 levels would anticipate the probability of achieving an iPTH in the range between 150 and 300 pg/ml during the efficacy assessment phase, were evaluated by logistic regression analysis. *P* values <0.05 were considered to represent statistical significance. All statistical analyses were performed using the SPSS software (version 18.0., SPSS Inc, Chicago, III, USA).

## Results

### Study population and baseline parameters

A total of 66 patients were enrolled and randomly assigned at 1:1 ratio - 33 were assigned to the cinacalcet group and 33 to the control group. Recruitment ended prematurely before target enrollment number (n=85) was reached, since the study underwent insufficiency of subjects who fulfilled the strict inclusion criteria. Baseline demographic characteristics, duration of dialysis, underlying disease, and renal Kt/V before randomization showed no statistical differences for both groups (Table 1). Furthermore, the baseline levels for iPTH, Ca, P, and Ca X P after vitamin D washout, and the FGF23 were similar between both groups. Six (18.2%) of the cinacalcet group and 11 (33.3%) of the control group had received active vitamin D (paricalcitol, calcitriol, or alfacalcidol) before screening. More than two-thirds of the patients in each group had received phosphate binders: 28 (84.8%) for the cinacalcet group and 25 (75.6%) for the control group.

**Table 1:** Patient demographics and baseline laboratory values

**Characteristic**	**Cinacalcet group (n = 33)**	**Control group (n = 33)**	***P*-value**
Sex, n (%)			0.324
Male	20 (60.6)	15 (45.5)	
Female	13 (39.4)	18 (54.5)	
Age, years			0.520
mean (SD)	48.8 (11.5)	47.2 (8.4)	
Range	25 to 71	28 to 64	
Duration of dialysis, months mean (SD)	78.7 (39.8)	71.3 (40.6)	0.454
Medical history, n (%)			
hypertension	32 (97.0)	29 (87.9)	0.355
diabetes mellitus	7 (21.2)	5 (15.2)	0.751
peripheral vascular disease	2 (6.1)	0 (0.0)	0.492
cerebrovascular disease	2 (6.1)	0 (0.0)	0.492
congestive heart failure	1 (3.0)	0 (0.0)	1.000
iPTH, median (Q1,Q3) (pg/ml)*	729 (518, 1424)	649 (490, 1143)	0.783
Serum calcium, mean (SD) (mg/dL)*	9.8 (0.6)	9.6 (0.8)	0.273
Serum phosphorus, mean (SD) (mg/dL)*	5.8 (1.7)	5.3 (1.4)	0.231
Ca X P, mean (SD) (mg^2^/dL^2^)*	53.8 (16.3)	48.7 (14.5)	0.189
Serum FGF23 (Q1,Q3) (RU/ml)*	3960 (2430, 6609)	2085 (741, 7182)	0.134
Renal K*t*/V (Q1, Q3)	0.0 (0.0, 0.1)	0.0 (0.0, 0.5)	0.170
Peritoneal K*t*/V (SD)	1.8 (0.5)	1.8 (0.6)	0.956
Total K*t*/V (SD)	1.9 (0.4)	2.1(0.6)	0.124

*Post-washout (week 0) value.

*Q1* first quartile, *Q3* third quartile.

Overall, twenty four (72.7%) of the cinacalcet group and thirty one (93.9%) of the control group completed the study. Reasons for dropouts included adverse reactions (n = 3 *vs.* 0, cinacalcet *vs*. control), withdrawal of consent (n = 3 *vs.* 1), kidney transplant (n = 2 *vs.* 1) and taking not allowed medication (n = 1 *vs.* 0). No subjects in the control group took cinacalcet throughout the study period.

### PTH and biochemical parameters

**Figure 2:**
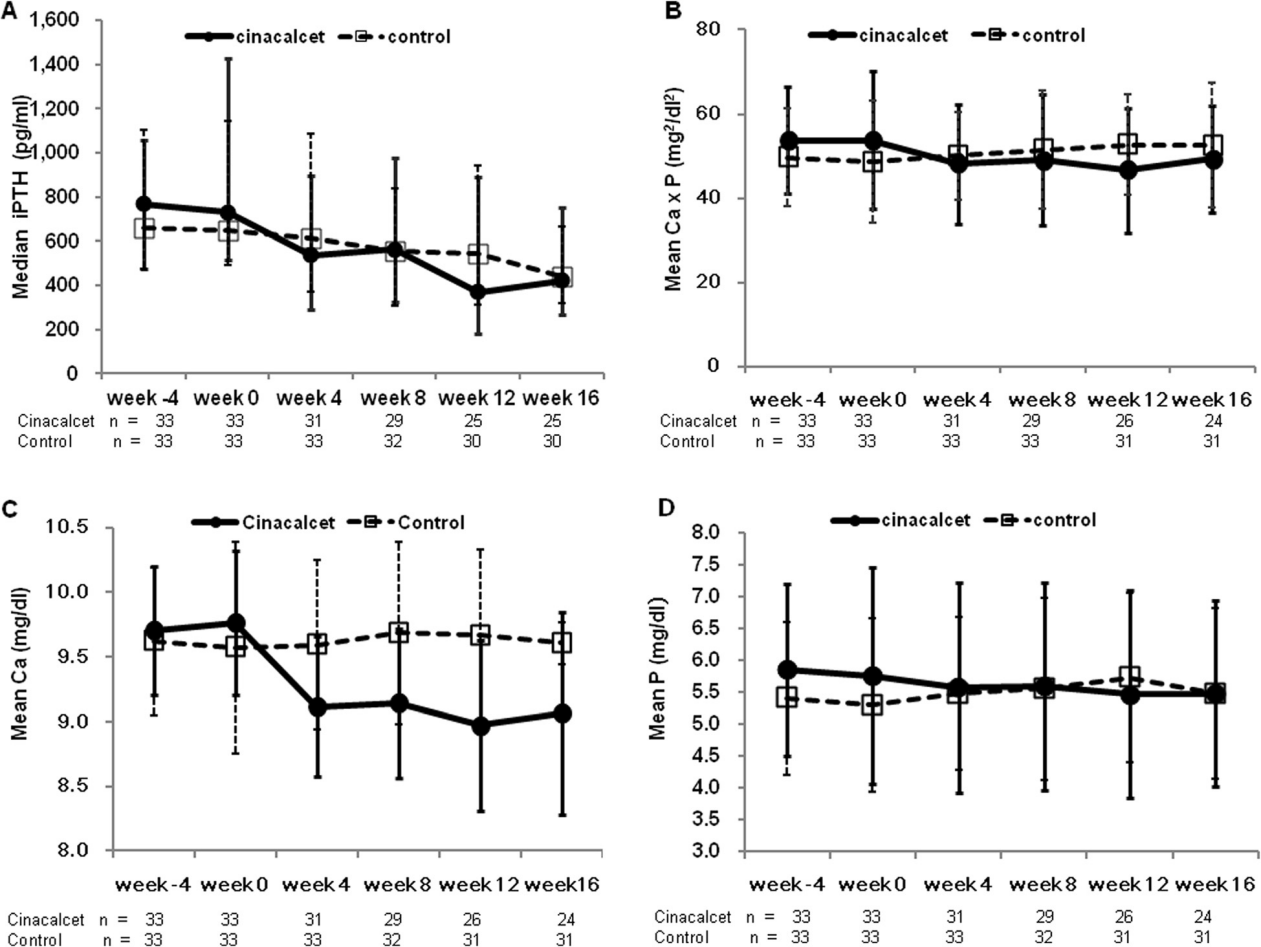
**Mineral metabolic parameters at each scheduled visit for both groups.** Median iPTH (**A**), mean calcium-phosphorus product (Ca X P) (**B**), calcium (Ca) (**C**), and phosphorus (P) (**D**). iPTH decreased by 42.3% in the cinacalcet group, and by 30.7% in the control group (*P* = 0.483). Error bars denote interquartile range (**A**), or standard deviation (SD) (**B**, **C**, **D**). iPTH, intact parathyroid hormone; Ca, calcium; P, phosphorus.

The median (Q1,Q3) iPTH decreased from 729 pg/ml (518, 1424 pg/ml) at baseline to 421 pg/ml (267, 753 pg/ml) during the efficacy assessment phase for the cinacalcet group, and from 649 pg/ml (490, 1143 pg/ml) to 441 pg/ml (317, 663 pg/ml) for the control group (Figure 2A). The median iPTH decreased by 42.3% in the cinacalcet group, and by 30.7% in the control group (*P* = 0.483). The proportion of patients who reached the primary endpoint (30% reduction in mean iPTH from the baseline) was not statistically different (48.5% *vs.* 51.5%, cinacalcet *vs.* control*, P* = 1.000). The proportion of patients who achieved an iPTH target between 150 and 300 pg/ml during the efficacy assessment phase was similar for both groups (24.3% *vs.* 18.2%, *P* = 0.764).

The proportion of patients who achieved Ca × P <55 mg^2^/dl^2^ (54.5% *vs.* 51.5%, *P* = 1.000), the Ca target (54.5% *vs.* 45.5%, *P* = 0.623), and P target (30.3% *vs.* 30.3%, P = 1.000) were similar. In the cinacalcet group, the Ca × P increased by 5.1% (*P* = 0.324 *vs.* control with 14.4%) and P increased by 8.3% in the cinacalcet group (*P* = 0.909 *vs.* control with 7.3%). Only the change of calcium was significantly different (−7.2% *vs.* 1.1%, cinacalcet *vs.* control*, P* <0.001).

The proportion of patients who achieved K/DOQI targets for iPTH, Ca, P, Ca × P, and their composite were not statistically different between two groups.

### Effect of cinacalcet on the FGF-23

**Figure 3:**
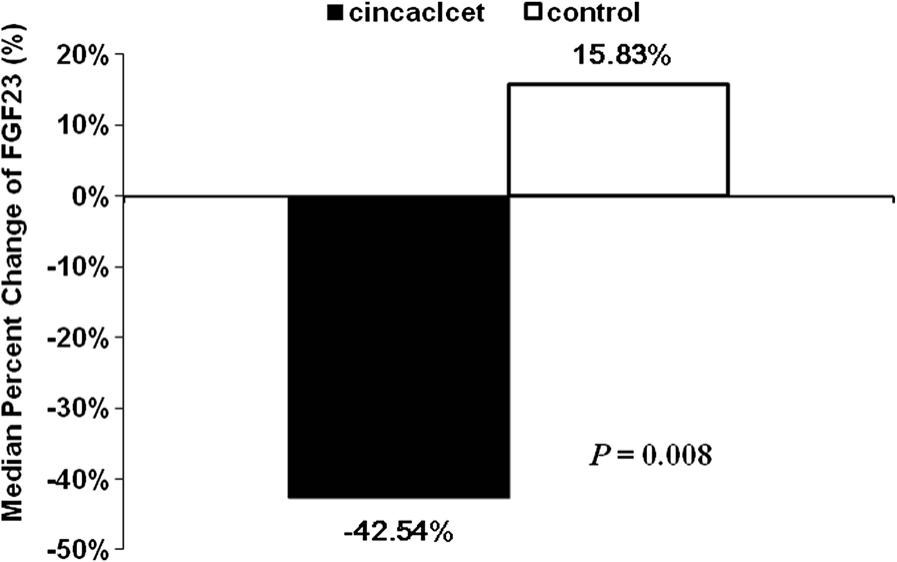
**Median percent change from baseline to final values of fibroblast growth factor (FGF23).** Cinacalcet group, n = 22; Control group, n = 30. The change of FGF23 was significantly different; -42.5% *vs.* 15.8%, *P* = 0.008 as compared with control group.

Cinacalcet group exhibited a significant decrease in FGF23 levels from 3,960 (2430, 6609) RU/ml at the baseline to 2,325 (514, 6598) RU/ml during the efficacy assessment phase (shown in median, Q1 and Q3), while the control group increased from 2,085 (741,7182) RU/ml to 2,415 (740,5191) RU/ml (*P* = 0.002). The change of FGF23 was significantly different (−42.5% *vs.* 15.8%, *P* = 0.008; Figure 3). Univariate and multivariate linear regression analyses were undertaken to explore factors correlated with the change of FGF23. In univariate linear regression, only cinacalcet treatment correlated significantly with the change of FGF23 (*P* = 0.004) while Ca, P, iPTH levels, and oral vitamin D dose did not (Table 2). After adjustment to age and sex, cinacalcet treatment remained an independent determinant of the serum FGF23 reduction (Table 3).

**Table 2:** Univariate linear regression analysis for association of the percent change in square root FGF23 with other variables

**Variable**	**B coefficient**	**95% CI**	***P*- value**
Sex (male *vs.* female)	3.80	−27.03 to 34.62	0.805
Age (per 1year)	0.51	−1.16 to 2.18	0.540
DM (yer *vs.* no)	24.36	−16.24 to 64.96	0.233
Treatment (cinacalcet *vs* control)	−42.45	−70.96 to −13.93	0.004
Vitamin D dose (per 1μg/day)	6.65	−49.86 to 63.15	0.814
Log-iPTH (pg/ml)*	0.27	−1.64 to 2.19	0.776
Ca (mg/dL)*	0.31	−1.40 to 2.02	0.718
P (mg/dL)*	−0.02	−0.46 to 0.41	0.916

* Percent change from baseline to assessment phase values was used for analysis.

*CI* confidence interval.

**Table 3:** Univariate and multivariate linear regression analysis for association of the percent change in square root FGF23 with treatment groups

	B coefficient* (95% CI)	***P*- value**
Univariate analysis	−42.45 (−70.96 to −13.93)	0.004
Multivariate analysis†	−43.58 (−73.07 to −14.09)	0.005

* Treatment effect was analyzed by cinacalcet versus control.

† adjusted for age and sex.

*CI* confidence interval.

Within the cinacalcet group, there was no significant difference in baseline FGF23 levels between the subjects who achieved iPTH target and those who did not [3870 RU/ml (1100, 5108 RU/ml) *vs.* 4159 RU/ml (2433, 7376 RU/ml), *P* = 0.427].

### Doses of medications and safety

**Figure 4:**
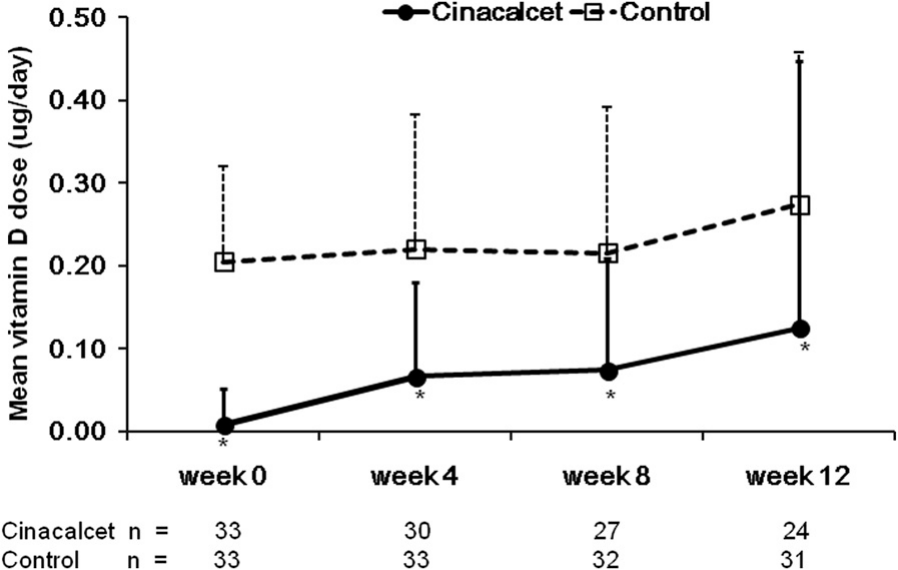
**Mean vitamin D dose at each time point.** Error bars denote standard deviation (SD). *P < 0.05 as compared with control group.

Cinacalcet group received 30.2 ± 18.0 mg/day of mean cinacalcet dose during the assessment phase (n = 24). The vitamin D doses during the assessment phase were 0.13 ± 0.32 μg/d (mean ± SD) in the cinacalcet group (n = 24, *P* <0.001) *vs.* 0.27 ± 0.18 μg/d (n = 31) in the control group (Figure 4). Phosphate binders were prescribed in 90.9% of the cinacalcet group *vs.* 81.8% (*P* = 0.475) of the control group at initiation of treatment and in 83.3% *vs.* 90.3% (*P* = 0.686) during assessment phase. During assessment phase, Ca-containing phosphate binders were prescribed for the 62.5% of the cinacalcet group and for the 48.4% of the control group. Ca-free phosphate binders were prescribed for 37.5% and 42.4%, respectively.

A total of 51.5% of the cinacalcet group and 33.3% of the control group experienced at least one adverse event. The most common adverse reactions were digestive symptoms. In the cinacalcet group, nausea/vomiting (18.2%), dyspepsia (3.0%), diarrhea (3.0%), and constipation (3.0%) were present. For the control group, nausea/vomiting, and diarrhea (3.0%, each) were most often observed. There were no significant differences in both groups. Other adverse reactions in the cinacalcet group were tingling sensation (6.1%), general weakness (3.0%), and headache (6.1%). There was no death or other serious adverse events in the entire study period in both groups. Severe hypocalcemia (serum corrected calcium <7.5 mg/dl at least once during the entire study period) occurred in one patient (3.0%) of each group, respectively. However, there was no symptomatic, or fatal hypocalcemia in both groups. All safety analyses were performed by intention-to-treat method.

## Discussion

The purpose of the present study was to investigate the effectiveness of cinacalcet-based *vs.* vitamin D based regimen in treating SHPT, achieving the K/DOQI targets for PD patients, and the cinacalcet effect on FGF23 suppression. The present study did not show a difference in the reduction of iPTH since it did not achieve the target enrollment number. However, the present study exhibited that serum FGF23 was significantly reduced during cinacalcet treatment. Moreover, cinacalcet effect on the FGF23 suppression was independent of its effect on serum iPTH, Ca, P, and the exposure to oral vitamin D.

Recent studies have shown that high levels of FGF23 are associated with poor outcomes such as left ventricular hypertrophy [15,16], progression of CKD [16] and increased overall mortality [17]. FGF23 could be a better prognosis predictor of cardiovascular disease than any other biomarkers. Therefore, reduction of FGF23 may become a novel target of treating SHPT for lowering cardiovascular risk and improving prognosis of CKD patients.

It is well documented in a few recent randomized clinical trials that cinacalcet is more efficient than conventional vitamin D based therapy in treating SHPT in hemodialysis patients [14,18,19]. Furthermore, *post hoc* analysis of these studies reported that cinacalcet modulated FGF23 reduction. However, the specific mechanism of cinacalcet effect on FGF23 suppression has not been elucidated. Therefore, it remains to be investigated whether cinacalcet lowers FGF23 *per se* or indirectly via modulating other mineral metabolic parameters. Several studies showed that the cinacalcet group received lower dose of vitamin D and exhibited lower level of PTH, compared with those receiving conventional vitamin D based therapy [14,18]. It is well known that PTH and vitamin D stimulate FGF23 production [20-22]. Therefore, one could postulate that the suppression of FGF23 in the cinacalcet group may be associated with lower exposure to vitamin D and lower level of PTH. Koizumi et al. [13] allowed relatively constant dosage of vitamin D during the study period. Since the dosage of vitamin D was allowed to be titrated according to the biochemical parameters in the present study, the cinacalcet group was prescribed with lower dose of vitamin D than the control group. However, in our univariate analysis, neither vitamin D dose nor other mineral metabolic parameters such as iPTH, Ca and P were associated with FGF23 change. Cinacalcet treatment was independently associated with FGF23 reduction, after adjustment to the age and sex. One could postulate that cinacalcet *per se* might have a modulatory effect on the FGF23 level.

The mechanism underlying the more direct association of FGF23 reduction with cinacalcet is yet unclear. FGF23 is synthesized and released by bone cells, mainly osteocytes [1,2]. Osteocyte and osteoblast were shown to express calcium sensing receptor (CaSR). Cinacalcet acts mainly by allosteric binding to the CaSR on the parathyroid cell membrane for regulating calcium homeostasis. Whether cinacalcet directly acts on the CaSR at the bone cells to regulate FGF23 secretion needs to be investigated.

The strengths of this study include prospective, randomized study and a design to follow change of FGF23 levels in PD patients with cinacalcet treatment, compared with conventional vitamin D treatment. Moreover, unlike other study reporting an effect of cinacalcet on the FGF23 reduction [13], the present study showed an independent association between cinacalcet treatment and FGF23 reduction. Unlike the EVOLVE trial, there was no possibility of cross-over of medication between the two arms, since cinacalcet was not commercially available in Korea during the study period [23].

However, the present study has a limitation of a small sample size and an open-labeled study. The investigators of the CUPID Study finished the recruitment prematurely before target number of enrollment was reached. This was because enrollment was delayed due to lack of subjects who fulfilled the strict inclusion criteria. This resulted in lack of statistical power to prove a difference in the iPTH reduction between two groups, which was the primary endpoint of the present study. Besides, the prescribed dose of cinacalcet was 30.2 ± 18.0 mg/day during assessment phase, which means our subjects received lower dose of cinacalcet than that reported in other studies [14,18]. This might be another explanation for the negative result as regards to the primary end point. Cinacalcet group exhibited more drop-outs that the control group. The analysis of FGF23 change was performed on a per-protocol basis. This may have resulted in a selection bias because only subjects who were more tolerable to the medication were included in the analysis. Dietary phosphorus intake, which is another determinant of serum FGF23 in CKD [24,25] could not be assessed in the present study. However, there were no significant differences the prescribed dose of oral phosphate binders and serum P levels between the two groups (data not shown). Therefore, we could postulate that the dietary P intake was similar between the two groups, although the present study did not analyze dietary phosphorus intake.

## Conclusions

The present study might suggest a more direct association between serum FGF23 and cinacalcet treatment, independent from other mineral metabolic parameters and vitamin D dose. Mechanistic research is warranted on the specific mechanism of FGF23 reduction by cinacalcet.

## Abbreviations

FGF23: Fibroblast growth factor 23; CUPID: Cinacalcet stUdy for Peritoneal Dialysis Patients In Double Arm on the Lowing Effect Of iPTH Level; NKF-K/DOQI: National kidney foundation kidney disease outcomes quality initiative; PD: Peritoneal dialysis; iPTH: Intact parathyroid hormone; CKD: Chronic kidney disease; SHPT: Secondary hyperparathyroidism; Ca: Calcium; P: Phosphorus; ELISA: Enzyme linked immunosorbent assay; IQR: Interquartile range; CaSR: Calcium sensing receptor; SD: Standard deviation; Q: Quartile; CI: Confidence interval; p.o: Per os.

## Competing interests

All authors declare that they have no competing interests.

## Authors’ contributions

HJK participated in the design of the study, reviewed and collected data using e-CRF and electronic medical records system, performed the statistical analysis, and drafted the manuscript. HK and NS participated in the analysis and interpretation of data. KYN, YLK, DK, JHC, YRS, and YHH participated in the design of the study, patient enrollment, acquisition of data, analysis, and interpretation of data. JL participated in analysis, and interpretation of data. YSK and CA participated in conception of the study, acquisition of data, and helped to draft the manuscript. KHO had made substantial contributions to conception and design of the study, and draft and revision of the manuscript. All authors read and approved the final manuscript.

## Pre-publication history

The pre-publication history for this paper can be accessed here:

http://www.biomedcentral.com/1471-2369/14/112/prepub

## References

[B1] Urakawa I Yamazaki Y Shimada T Iijima K Hasegawa H Okawa K Fujita T Fukumoto S Yamashita T Klotho converts canonical FGF receptor into a specific receptor for FGF23 Nature 2006 444 770 774 10.1038/nature05315 17086194

[B2] Kuro-o M Matsumura Y Aizawa H Kawaguchi H Suga T Utsugi T Ohyama Y Kurabayashi M Kaname T Kume E Iwasaki H Iida A Shiraki-Iida T Nishikawa S Nagai R Nabeshima YI Mutation of the mouse klotho gene leads to a syndrome resembling ageing Nature 1997 390 45 51 10.1038/36285 9363890

[B3] Saito H Kusano K Kinosaki M Ito H Hirata M Segawa H Miyamoto K Fukushima N Human fibroblast growth factor-23 mutants suppress Na+−dependent phosphate co-transport activity and 1alpha,25-dihydroxyvitamin D3 production J Biol Chem 2003 278 2206 2211 10.1074/jbc.M207872200 12419819

[B4] Shimada T Hasegawa H Yamazaki Y Muto T Hino R Takeuchi Y Fujita T Nakahara K Fukumoto S Yamashita T FGF-23 is a potent regulator of vitamin D metabolism and phosphate homeostasis J Bone Miner Res 2004 19 429 435 15040831 10.1359/JBMR.0301264

[B5] Isakova T Wahl P Vargas GS Gutierrez OM Scialla J Xie H Appleby D Nessel L Bellovich K Chen J Hamm L Gadegbeku C Horwitz E Townsend RR Anderson CA Lash JP Hsu CY Leonard MB Wolf M Fibroblast growth factor 23 is elevated before parathyroid hormone and phosphate in chronic kidney disease Kidney Int 2011 79 1370 1378 10.1038/ki.2011.47 21389978 PMC3134393

[B6] Gutierrez OM Januzzi JL Isakova T Laliberte K Smith K Collerone G Sarwar A Hoffmann U Coglianese E Christenson R Wang TJ deFilippi C Wolf M Fibroblast growth factor 23 and left ventricular hypertrophy in chronic kidney disease Circulation 2009 119 2545 2552 10.1161/CIRCULATIONAHA.108.844506 19414634 PMC2740903

[B7] Hsu HJ Wu MS Fibroblast growth factor 23: a possible cause of left ventricular hypertrophy in hemodialysis patients Am J Med Sci 2009 337 116 122 10.1097/MAJ.0b013e3181815498 19214027

[B8] Seiler S Cremers B Rebling NM Hornof F Jeken J Kersting S Steimle C Ege P Fehrenz M Rogacev KS Scheller B Bohm M Flister D Heine GH The phosphatonin fibroblast growth factor 23 links calcium-phosphate metabolism with left-ventricular dysfunction and atrial fibrillation Eur Heart J 2011 32 2688 2696 10.1093/eurheartj/ehr215 21733911

[B9] Mirza MA Larsson A Melhus H Lind L Larsson TE Serum intact FGF23 associate with left ventricular mass, hypertrophy and geometry in an elderly population Atherosclerosis 2009 207 546 551 10.1016/j.atherosclerosis.2009.05.013 19524924

[B10] Fliser D Kollerits B Neyer U Ankerst DP Lhotta K Lingenhel A Ritz E Kronenberg F Kuen E Konig P Kraatz G Mann JF Muller GA Kohler H Riegler R Fibroblast growth factor 23 (FGF23) predicts progression of chronic kidney disease: the mild to moderate kidney disease (MMKD) study J Am Soc Nephrol 2007 18 2600 2608 10.1681/ASN.2006080936 17656479

[B11] Isakova T Xie H Yang W Xie D Anderson AH Scialla J Wahl P Gutierrez OM Steigerwalt S He J Schwartz S Lo J Ojo A Sondheimer J Hsu CY Lash J Leonard M Kusek JW Feldman HI Wolf M Fibroblast growth factor 23 and risks of mortality and end-stage renal disease in patients with chronic kidney disease JAMA 2011 305 2432 2439 10.1001/jama.2011.826 21673295 PMC3124770

[B12] Wetmore JB Liu S Krebill R Menard R Quarles LD Effects of cinacalcet and concurrent low-dose vitamin D on FGF23 levels in ESRD Clin J Am Soc Nephrology 2010 5 110 116 10.2215/CJN.03630509 PMC2801647 19965548

[B13] Koizumi M Komaba H Nakanishi S Fujimori A Fukagawa M Cinacalcet treatment and serum FGF23 levels in haemodialysis patients with secondary hyperparathyroidism Nephrol Dial Transplant 2012 27 784 790 10.1093/ndt/gfr384 21730210

[B14] Fishbane S Shapiro WB Corry DB Vicks SL Roppolo M Rappaport K Ling X Goodman WG Turner S Charytan C Cinacalcet HCl and concurrent low-dose vitamin D improves treatment of secondary hyperparathyroidism in dialysis patients compared with vitamin D alone: the ACHIEVE study results Clin J Am Soc Nephrol 2008 3 1718 1725 10.2215/CJN.01040308 18945995 PMC2572296

[B15] Kirkpantur A Balci M Gurbuz OA Afsar B Canbakan B Akdemir R Ayli MD Serum fibroblast growth factor-23 (FGF-23) levels are independently associated with left ventricular mass and myocardial performance index in maintenance haemodialysis patients Nephrol Dial Transplant 2011 26 1346 1354 10.1093/ndt/gfq539 20813767

[B16] Seiler S Heine GH Fliser D Clinical relevance of FGF-23 in chronic kidney disease Kidney Int Supplement 2009 114 S34 S42 10.1038/ki.2009.405 19946326

[B17] Gutierrez OM Mannstadt M Isakova T Rauh-Hain JA Tamez H Shah A Smith K Lee H Thadhani R Juppner H Wolf M Fibroblast growth factor 23 and mortality among patients undergoing hemodialysis N Engl J Med 2008 359 584 592 10.1056/NEJMoa0706130 18687639 PMC2890264

[B18] Messa P Macario F Yaqoob M Bouman K Braun J von Albertini B Brink H Maduell F Graf H Frazao JM Bos WJ Torregrosa V Saha H Reichel H Wikie M Zani VJ Molemans B Carter D Locatelli F The OPTIMA study: assessing a new cinacalcet (Sensipar/Mimpara) treatment algorithm for secondary hyperparathyroidism Clin J Am Soc Nephrol 2008 3 36 45 10.2215/CJN.03591006 18178780 PMC2390975

[B19] Sprague SM Evenepoel P Curzi MP Gonzalez MT Husserl FE Kopyt N Sterling LR Mix C Wong G Simultaneous control of PTH and CaxP Is sustained over three years of treatment with cinacalcet HCl Clin J Am Soc Nephrol 2009 4 1465 1476 10.2215/CJN.06141108 19696213 PMC2736698

[B20] Liu S Tang W Zhou J Stubbs JR Luo Q Pi M Quarles LD Fibroblast growth factor 23 is a counter-regulatory phosphaturic hormone for vitamin D J Am Soc Nephrol 2006 17 1305 1315 10.1681/ASN.2005111185 16597685

[B21] Nishi H Nii-Kono T Nakanishi S Yamazaki Y Yamashita T Fukumoto S Ikeda K Fujimori A Fukagawa M Intravenous calcitriol therapy increases serum concentrations of fibroblast growth factor-23 in dialysis patients with secondary hyperparathyroidism Nephron Clin Pract 2005 101 c94 c99 10.1159/000086347 15956805

[B22] Donate-Correa J Muros-de-Fuentes M Mora-Fernandez C Navarro-Gonzalez JF FGF23/Klotho axis: phosphorus, mineral metabolism and beyond Cytokine Growth Factor Rev 2012 23 37 46 10.1016/j.cytogfr.2012.01.004 22360923

[B23] Chertow GM Block GA Correa-Rotter R Drueke TB Floege J Goodman WG Herzog CA Kubo Y London GM Mahaffey KW Mix TC Moe SM Trotman ML Wheeler DC Parfrey PS Effect of cinacalcet on cardiovascular disease in patients undergoing dialysis N Engl J Med 2012 367 2482 2494 23121374 10.1056/NEJMoa1205624

[B24] Ferrari SL Bonjour JP Rizzoli R Fibroblast growth factor-23 relationship to dietary phosphate and renal phosphate handling in healthy young men J Clin Endocrinol Metab 2005 90 1519 1524 15613425 10.1210/jc.2004-1039

[B25] Antoniucci DM Yamashita T Portale AA Dietary phosphorus regulates serum fibroblast growth factor-23 concentrations in healthy men J Clin Endocrine Metab 2006 91 3144 3149 10.1210/jc.2006-0021 16735491

